# A Dual-Bonded Approach for Improving Hydrogel Implant Stability in Cartilage Defects

**DOI:** 10.3390/ma10020191

**Published:** 2017-02-16

**Authors:** Yan Liu, Yuxuan Wu, Lei Zhou, Zhengao Wang, Cong Dai, Chengyun Ning, Guoxin Tan

**Affiliations:** 1 Institute of Chemical Engineering and Light Industry, Guangdong University of Technology, Guangzhou 510006, China; liuyan0921@163.com (Y.L.); daicong0312@163.com (C.D.); 2 Department of Electronic Communication & Software Engineering, Nanfang College of Sun Yat-sen University, Guangzhou 510970, China; ulvelaik@outlook.com; 3 School of Materials Science and Engineering, South China University of Technology, Guangzhou 510641, China; zhoulei7170@126.com (L.Z.); mszhengao@mail.scut.edu.cn (Z.W.)

**Keywords:** titanium, chondroitin sulphate, cartilage tissue, aldehyde, adhesion

## Abstract

Integration and stability of hydrogels and surrounding cartilage/bone tissue is crucial for both immediate functionality and long-term performance of the tissue. In this work, chondroitin sulphate (CS) a polysaccharide found in cartilage and other tissues was used to synthesize a tough hydrogel that was chemically functionalized with methacrylate and aldehyde groups, bonding to surrounding tissue via a dual-bonded approach. The hydrogel can not only chemically anchor onto implanted titanium at the subchondral bone, but also on cartilage tissue via the Schiff-base reaction. In vitro experiments confirmed that the strategy improved hydrogel implant stability with cartilage tissue, was favorable for chondrocyte attachment, and has the potential to quickly and effectively repair cartilage defects and maintain joint functionality for a long time.

## 1. Introduction

Articular cartilage is mainly composed of collagen and proteogly can and other special connective tissues. Without blood vessels, nerves, and lymphoid tissues, once there is injury to the articular cartilage, it has almost no effective ability to repair and rebuild itself [1]. Hydrogels are a kind of three-dimensional porous network with similar mechanical characteristics to those of articular cartilage [2,3,4]. As an ideal material substitute to repair articular cartilage, the physical and chemical properties of hydrogels such as their excellent biocompatibility have received extensive attention from researchers [5,6]. However, compared to the extremely tough bonding of cartilage to bone, the bonding of synthetic hydrogel–bone and synthetic hydrogel–cartilage interfaces are relatively weak and brittle [7]. Thus, there is a significant need for a tissue integration method for cartilage repair using hydrogels.

Chondroitin sulphate (CS) is a polysaccharide found in cartilage and other tissues in the body [8]. CS has a series of excellent biological properties for tissue integration, such as immunomodulatory and anti-inflammatory effects [9], and water and nutrient absorption [10]. In this study, it was hypothesized that using CS to simulate the natural composition of cartilage polysaccharides and the extracellular matrix would yield a more similar microenvironment for the growth of cartilage cells. CS was chemically functionalized with both methacrylate and aldehyde groups in order to form two functional groups: one group to covalently bond to the nail of implanted titanium at the subchondral bone; the second group bonding to the surrounding cartilage. In prior work, we developed a design strategy that could provide a tough bonding of cross-linked hydrogel networks to titanium surfaces in an aqueous environment, using silane functionalization chemistry [11]. Herein, we hypothesize that integration between CS and bone can be achieved using a silane-modified titanium implant. The aldehyde groups in the hydrogel can react with the amine groups present on the cartilage tissue surface, which significantly improved the bonding strength of the biomaterial with the surrounding cartilage [12,13]. The dual-bonded approach has the potential to quickly and effectively repair cartilage defects and maintain joint function for a long time.

## 2. Experimental Section

### 2.1. Material Preparation

Titanium plates (purity 99.5%, 100–200 μm thickness) were obtained from Baoji Qichen New Material Technology Co., Ltd. (Xi’an, China). 3-(Trimethoxysilyl) propyl-methacrylate (TMSPMA, 98%) was purchased from Sigma (Saint Louis, MO, USA), and was used as received. Chondroitin 4-sulfate sodium salt (lyophilized powder, cell culture, ≥85%, Aladdin, Shanghai, China), methacrylate (20 mL, 98% purity); Actin-Tracker Green (Beyotime, Shanghai, China), 4′,6-diamidino-2-phenylindole (DAPI, Sigma), calcein AM, and propidium iodide (PI) were obtained from Sigma-Aldrich (Saint Louis, MO, USA). ATDC-5 chondrocyte cells were provided by the American Type Culture Collection (Manassas, VA, USA). All other chemicals were of analytical grade and used without further purification.

### 2.2. Titanium Surface Silicon Alkylation Treatment

The Ti samples were disposed to Ti-TMSPMA, according to a previously reported method [11]. The Ti samples were ultrasonically cleaned in distilled water, acetone, and ethanol, and then activated by soaking in 10% NaOH solution at 80 °C, overnight (Ti-OH). Subsequently, the oxidized titanium samples were immersed in a 10% TMSPMA solution at 80 °C, overnight. Then, the samples were rinsed with alcohol and dried with a nitrogen stream flow. These samples were referred to as Ti-TMSPMA or Ti’.

### 2.3. Preparation for Chondroitin Sulphate–Methacrylate Aldehyde Macromer

CS (2 g) was dissolved in 80 mL of phosphate-buffered saline (PBS, pH = 7.4), followed by the addition of methacrylate (20 mL). The reaction solution was vigorously stirred at room temperature for 2 h, and then transferred to 4 °C for 24 h. The macromer was precipitated with alcohol several times and dried under vacuum for 48 h (CS-MA). The resulting CS-MA (300 mg) was oxidized with sodium periodate (350 mg, Aladdin) in 5 mL of deionized water for 20 h, in the dark, under vigorous stirring. The product chondroitin sulphate methacrylate aldehyde (CS-MA aldehyde) was then purified using alcohol and stored at −20 °C.

### 2.4. Bonding Hydrogel to the Ti-TMSPMA Substrates

In these experiments, CS-MA and CS-MA aldehyde macromonomer solution in PBS (5%, *w*/*v*) containing the photoinitiator 2-hydroxy-l-[4-(hydroxyethoxy)phenyl]-2-methyl-lpropanone (Irgacure 2959, 0.5% *w*/*v*), was added to TMSPMA-modified titanium substrates and immediately covered with a piece of glass coated with octadecyltrichlorosilane (OTS, 97.5%), followed by irradiation with ultraviolet light for 25 s (UV intensity of 700 mW/cm^2^). After UV illumination, the modified hydrogel was covalently immobilized onto the substrate’s surface. Then, the samples were frozen at −80 °C for 8 h and were then removed from −80 °C and quickly placed in a lyophilizer for three days (Ti’-CS-MA or Ti’-CS-MA aldehyde).

### 2.5. In Vitro Mechanical Stability Study

The in vitro mechanical stabilities of the hydrogel layers on the substrates were determined via ultrasonication tests. The cross-linked CS-MA hydrogel layers which were covalently bonded to TMSPMA-modified titanium substrates, and the hydrogel layers bonded to non-TMSPMA-assembled substrates were tested via ultrasonication (Kunshan Ultrasonic Instruments Co. Ltd., Kunshan, China, 40 kHz, 80 W) for 5 min. The samples were then frozen at −80 °C for 6 h and then removed and quickly placed in a lyophilizer for three days for SEM characterization. Twenty microliters of 5.0 wt % CS-MA hydrogel (aldehyde or without) were formed in situ on pork cartilage tissue in UV irradiation experiments. Breakage or detachment between the hydrogel and tissue after twisting was seen in photographs.

### 2.6. Characterizations

^1^H-NMR and Fourier transform infrared (FTIR) spectroscopy were performed on a Bruker AVANCE III 400 MHz Superconducting Fourier (Bruker, Billerica, MA, USA) and a Thermo Scientific Nicolet IS10 spectrometer (Thermo, Waltham, MA, USA), respectively. Surface silicon alkylation was characterized using X-ray photoelectron spectroscopy (XPS, Phi X-tool instrument, Osaka, Japan). Morphological studies of the porous hydrogels were conducted using scanning electron microscopy (SEM; ZEISS Ultra 55, Zeiss, Oberkochen, Germany).

### 2.7. Cell Adhesion and Proliferation Assays

ATDC-5 cartilage cells cultured for 24 h on pure Ti and Ti’-CS-MA, and Ti’-CS-MA aldehyde hydrogel substrates were stained with calcein (live, 1 mg/mL) and propidium iodide (dead, 1 mg/mL) to evaluate cell viability. The cells were seeded and adhered to the bottom of a 48-well plate at 1.0 × 10^4^ cells/well. After incubation for 40 min at 37 °C, the samples were washed with PBS and imaged using an Olympus IX73 inverted microscope equipped with an Olympus U-HGLGPS fluorescence illumination source and an Olympus XM10 camera (Shinjuku, Tokyo, Japan). For cell adhesion studies, cartilage cell adhesion on the substrates for 24 h was observed by staining with Actin-Tracker Green and DAPI at 1.0 × 10^4^ cells/well in 48-well plates. Fluorescent images of the cells were observed using laser co-focus light microscopy. Cell proliferation was examined using Cell Counting Kit-8 assays (CCK-8, Dojindo Laboratories, Rockville, MD, USA) after incubation for 1, 3, and 7 d. The medium was removed, and the samples were washed with PBS. Subsequently, 500 μL of fresh medium containing 10% CCK-8 reagent was added to each sample, which was then incubated at 37 °C for 3 h under the same standard culture conditions. Then, optical density (OD) at 490 nm was measured using a microplate reader (*n* = 4).

### 2.8. Statistical Analyses

All experiments were conducted at least three times, and all values are reported as mean ± standard deviation. Differences between the two sets of data were considered significant when ** *p* < 0.01.

## 3. Results and Discussion

**Figure 1:**
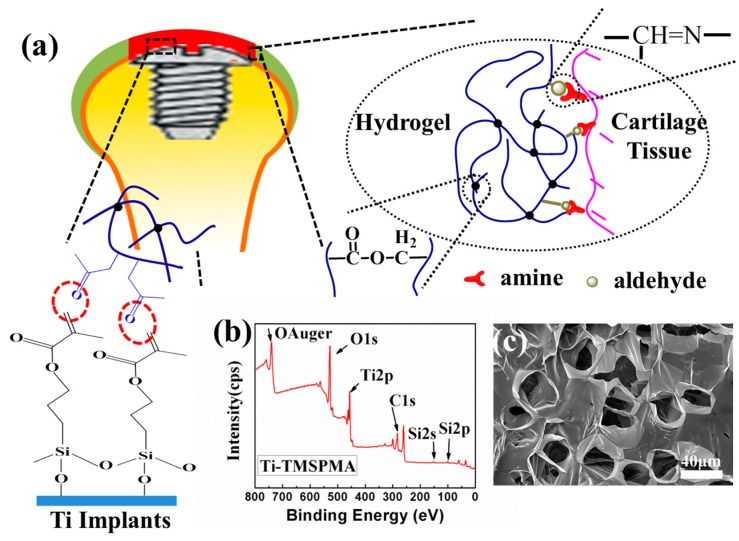
(**a**) Schematic illustration of the mechanisms for chondroitin sulphate methacrylate(CS-MA) aldehyde hydrogel construction on titanium, and simultaneous tissue integration; (**b**) X-ray photoelectron spectroscopy (XPS) overview spectra of Ti-TMSPMA; (**c**) Low-magnification and high-magnification scanning electron microscope (SEM) images of the CS-MA aldehyde hydrogel on Ti-TMSPMA.

As shown in Figure 1a, once the 3-(trimethoxysilyl) propyl-methacrylate (TMSPMA) molecules grafted to the titanium surface, the CS-MA aldehyde monomers were subsequently able to form a crosslinked hydrogel network via a radical reaction using UV illumination, double bonds, aldehyde, and acrylate (within dotted ellipses) [14]. CS-MA aldehyde was chemically multifunctionalized with both aldehyde and methacrylate groups in order to form two functional arms. One arm was covalently bonded to the biomaterial implant’s surface, and the second arm was bonded to the host tissue surface. The randomly-distributed aldehyde functionality of the oxidized CS was conjugated with amines present on the tissue surfaces via the Schiff-base reaction [13]. The surface chemical composition after processing with TMSPMA was further determined using X-ray photoelectron spectroscopy (XPS). After the silanization reaction, the presence of the Si2p signals at 102.1 eV (Figure 1b) demonstrated that the silane molecules had been successfully introduced to the Ti surface as a TMSPMA molecular bridge. As can be seen in Figure 1c, the resulting structure was a classic porous network structure of hydrogels. The binding force between the molecular bridge and the hydrogel coatings has been previously validated by our team [11], and will not be covered here.

**Figure 2:**
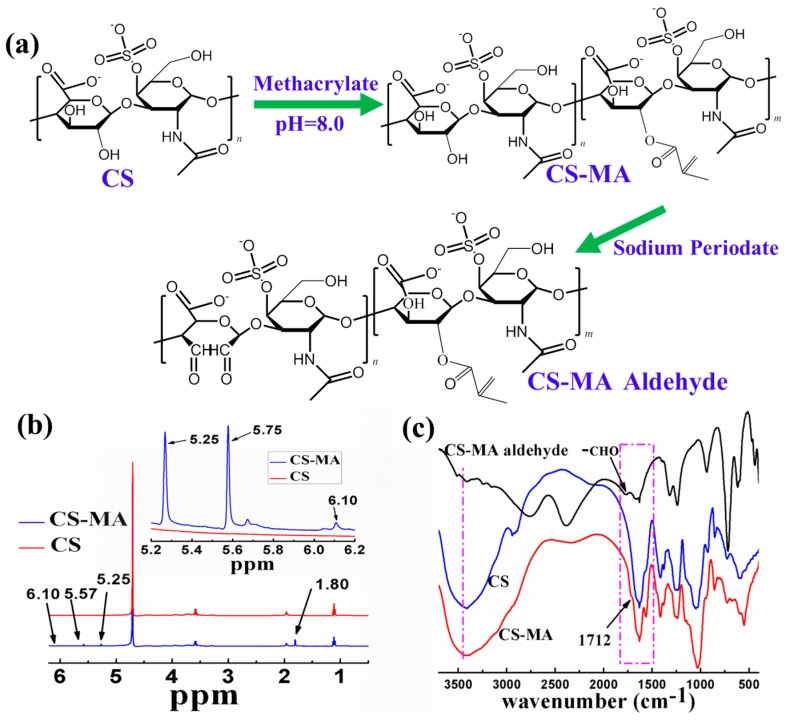
(**a**) Synthetic pathway of methacrylated chondroitin sulfate (CS-MA) and CS-MA aldehyde; (**b**) Structure characterization of CS and CS-MA using ^1^H-NMR (400 MHz); (**c**) Fourier-transform infrared (FTIR) spectra of CS, CS-MA, and CS-MA aldehyde.

In this work, chondroitin sulphate was first conjugated to the methacrylate groups in order to form methacrylated chondroitin sulphate (CS-MA) via the dehydration–condensation reaction [15,16]. Based on Le Châtelier’s principle, the reaction was driven in the right direction by neutralizing methacrylic acid with sodium hydroxide. Second, adjacent hydroxyls on the chondroitin sulphate backbone were oxidized with sodium periodate in order to form aldehyde groups, as shown in Figure 2a. The evidence of MA incorporation in CS was observed using ^1^H-NMR (Figure 2b). Two distinct peaksat 5.25 and 5.65 ppm (a small number was at6.1 ppm) were attributed to the two protons attached to the double bond (C=CH_2_). The peak shown at 1.80 ppm was ascribed to the methyl groups adjacent to the double bond (CH_3_-C=CH_2_), which are not present in virgin chondroitin sulfate. As shown in Figure 2c, a characteristic ester absorption band appeared at 1712 cm^−1^. After treatment by oxidation, the hydroxyl groups on the CS decreased significantly; an aldehyde peak appeared at 1765 cm^−1^. The successful substitution of MA in CS was again confirmed using FTIR.

**Figure 3:**
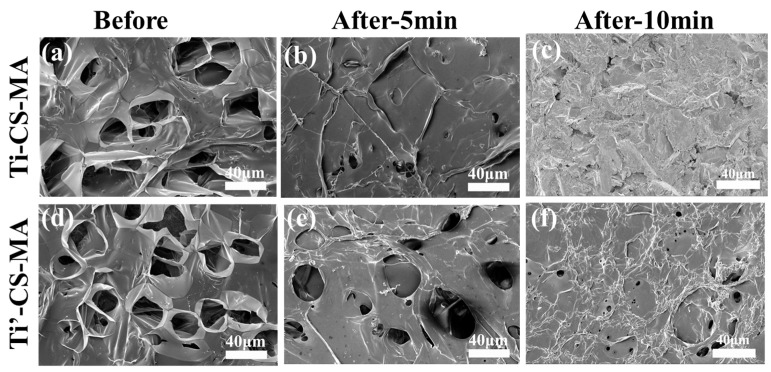
(**a**) SEM images of the cross-linked hydrogel layers that were covalent to the Ti surface (Ti-CS-MA,) before and after (**b**) exposure to ultrasonication (40 kHz, 80 W) for 5 min. After 10 min, the hydrogel layer was cut from the titanium surface, with only the pure titanium remaining (**c**). The hydrogel layers covalent to the Ti-TMSPMA substrates ((Ti’-CS-MA, (**d**)) maintained a good coating morphology after ultrasonication (40 kHz, 80 W) for 5 min (**e**), and no detectable damage was noted compared to non-TMSPMA-assembled titanium substrates. However, apparent damage was seen after exposure to ultrasonication (40 kHz, 80 W) for 10 min (**f**).

**Figure 4:**
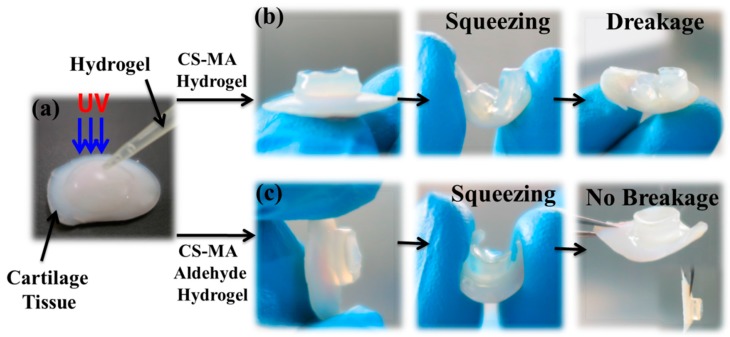
(**a**) Photographs of the (200 μL, 5.0 wt %) in situ hydrogel are shown on pork cartilage tissue upon UV irradiation; (**b**) CS-MA hydrogel; (**c**) CS-MA aldehyde hydrogel.

Furthermore, hydrogels with cartilage tissue may be required in tissue engineering applications in order to achieve the appropriate mechanical properties. As shown in Figure 3, the cross-linked hydrogel layers that were covalent to the Ti-TMSPMA substrates exhibited a greater stability than the non-TMSPMA-assembled substrates after strong ultrasonication (40 kHz, 80 W) for 5 min. Damage to the hydrogel layer morphology was observed, and the porous structure had almost disappeared (Figure 3a,c); however, the hydrogel layers that were covalent to the Ti-TMSPMA substrates maintained a good coating morphology, and detectable damage was not seen (Figure 3b,d), which proved the stability between the hydrogels and base-material enhancement after grafting [17]. The hydrogel layers covalent to the Ti-TMSPMA after exposure to ultrasonication (40 kHz, 80 W) for 10 min (Figure 3e) were obviously damaged. From the photographs of the CS-MA aldehyde hydrogel (with aldehyde or without) formed in situ on pork cartilage tissue in UV irradiation experiments (as shown in Figure 4) after twisting, there was no sign of breakage or detachment of the aldehyde hydrogel, suggesting the considerable tissue binding strength of our hydrogel. No breakage or detachment between the hydrogel and tissue after twisting is seen in the photographs.

**Figure 5:**
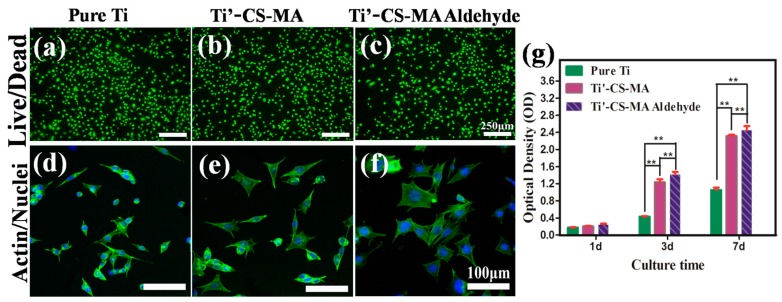
Live/dead staining of ATDC-5 cartilage cells on (**a**) pure Ti; (**b**) Ti’-CS-MA; (**c**) Ti’-CS-MA aldehyde hydrogel surface. Scale bar: 250 μm. Morphology of ATDC-5 cartilage cells on (**d**) pure Ti; (**e**) Ti’-CS-MA; (**f**) Ti’-CS-MA aldehyde surface. Scale bar: 100 μm. (**g**) Cell viability of each sample after 1, 3, and 7 d. ** *p* < 0.01.

In addition, to illustrate the growth behavior of cartilage cells on various surfaces, fluorescence images of ATDC-5 cartilage cells were recorded after culturing for 24 h in order to evaluate cytotoxicity (Figure 5a–c). Image Pro Plus software was used to calculate the average cell survival rates of the three samples. For (a) pure Ti; (b) Ti’-CS-MA; and (c) Ti’-CS-MA aldehyde hydrogel, the average cell survival rates were 98.5% ± 1.2%, 98.8% ± 0.5%, and 99.2% ± 0.8%, respectively. The results showed high cell survival rates of the cartilage cells after culturing for 24 h, and the three materials for the cells were non-toxic. ATDC-5 cartilage cells were then cultured on the substrates, and all three samples (shown in Figure 5d–f) exhibited spreading and adhesion morphologies. However, cells spread out best on the CS-MA aldehyde hydrogel surface; CS-MA hydrogel was the next best option. Cartilage cell viability was observed for 1, 3, and 7 d, using Cell Counting Kit-8 assays (Figure 5g). Compared with pure Ti, the cell growth rate after 3 d and 7d had significantly increased viability of cells on Ti’-CS-MA and Ti’-CS-MA aldehyde hydrogel (** *p* < 0.01). The cells on Ti’-CS-MA aldehyde hydrogel grew better than on the Ti’-CS-MA hydrogel. Thus, the results suggested that the CS-MA/aldehyde hydrogel still exhibited cell viability until seven days.

## 4. Conclusions

A chondroitin sulphate–methacrylate aldehyde hydrogel on a titanium surface was successfully fabricated for biomaterial cartilage tissue integration. The methacrylate and aldehyde groups can react with titanium substrates and amines on cartilage tissue, respectively. This approach led to the mechanical stability of the hydrogel prosthesis in cartilage defects. Simulating the natural composition of cartilage polysaccharides and the extracellular matrix can quickly and effectively repair cartilage defects and maintain joint functionality for a long time.
